# OCT and OCT-A biomarkers in multiple sclerosis - review

**DOI:** 10.22336/rjo.2023.20

**Published:** 2023

**Authors:** Mihai Bostan, Ruxandra Pîrvulescu, Cristina Tiu, Inna Bujor, Alina Popa-Cherecheanu

**Affiliations:** * “Carol Davila” University of Medicine and Pharmacy, Bucharest, Romania; ** Ophthalmology Emergency Hospital, Bucharest, Romania; *** Department of Ophthalmology, University Emergency Hospital, Bucharest, Romania; **** Department of Neurology, University Emergency Hospital, Bucharest, Romania

**Keywords:** multiple sclerosis, optical coherence tomography, optical coherence tomography angiography

## Abstract

**Objective:** Retinal neuronal and vascular changes have been observed in multiple sclerosis (MS) patients. The aim of this review was to highlight the most current optical coherence tomography (OCT) and optical coherence tomography angiography (OCT-A) data in MS and to provide information about the possibility of using OCT / OCT-A parameters as biomarkers for screening, diagnosis and monitoring of MS.

**Methods:** To carry out this review, a meticulous literature search was undergone on PubMed between 2014 and the present day, using the following terms: “multiple”, “sclerosis”, “optical”, “coherence”, “tomography” and “angiography”. Additional studies were found via references, being chosen according to relevance.

**Results:** Retinal nerve fiber layer (RNFL) and ganglion cell layer (GCL) were significantly lower in MS patients compared to controls, and correlated with clinical and paraclinical variables, such as visual function, disability, and magnetic resonance imaging (MRI). Retinal capillary plexuses could be higher, lower or the same, and the best OCT-A microvasculature parameter for the detection of MS was the superficial capillary plexus (SCP). The reduced retinal vessel density (VD) was correlated with the disability in MS.

**Conclusions:** OCT and OCT-A parameters could improve the development of retinal biomarkers for screening, early diagnosis and monitoring the disease progression of MS, and they could improve the development of potential future therapies that could slow or stop the course of this incurable disease.

**Abbreviations: **DCP = deep capillary plexus; EDSS = Expanded Disability Status Scale; GCC = ganglion cell complex; GCL = ganglion cell layer; MRI = magnetic resonance imaging; MS = Multiple sclerosis; OCT = optical coherence tomography; OCT-A = optical coherence tomography angiography; ON = optic neuritis; RNFL = retinal nerve fiber layer; SCP = superficial capillary plexus; VD = vessel density

## Introduction

Multiple sclerosis (MS) is a chronic autoimmune disease of the central nervous system that typically presents in young people [**1**]. Considering the eye as the brain’s window [**2**] and given the optically accessible eye, the structural and vascular changes in the retina may act as a surrogate for the brain changes in MS [**3**]. 

Optical coherence tomography (OCT) is a non-invasive reproducible method that has significantly developed over the past 20 years [**4**,**5**]. The OCT technology is comparable to that of the ultrasound, but it requires infrared light to analyze the backscattering of retinal tissues [**5**]. Using patterns of infrared reflection, OCT allows a precise quantification of the retinal layers, such as retinal nerve fiber layer (RNFL) and ganglion cell layer (GCL) [**5**]. One of the most significant pathogenic components that leads to persistent disability in MS is neurodegeneration and OCT has proven to be a sensitive and useful alternative to magnetic resonance imaging (MRI) for the investigation of this process [**5**]. 

Optical coherence tomography angiography (OCT-A) is a new diagnostic tool, which was commercially introduced in 2014 [**6**] and may provide the chance to evaluate modifications in cerebral vasculature by looking at retinal vasculature [**2**]. OCT-A makes it possible to see the retinal capillary layers and choroidal vascular network in vivo [**7**]. 

The application of OCT and OCT-A in MS is still unclear, but it seems promising [**8**]. Given the difficulty of MS diagnosis, which is based on an expensive and time-consuming method, there is a substantial interest to utilize the OCT and OCT-A parameters as low-cost biomarkers for screening, diagnosis and monitoring of MS [**9**].

## Methods

To carry out this review, a meticulous literature search was undergone on PubMed using the following terms: “multiple”, “sclerosis”, “optical”, “coherence”, “tomography” and “angiography”. The inclusion criteria were: articles that mentioned the terms above and studies that were performed between 2014 and the present day, after the commercially introduction of OCT-A [**6**]. 

Additional studies were found via references, and were chosen based on relevance. 

## Results

According to current literature, it is well known that MS has statistically significant reductions in the thickness of the RNFL and the GCL [**8**,**10**,**11**], even in the absence of prior optic neuritis (ON) [**12**]. A newly compensated peripapillary RNFL thickness for ocular anatomical parameters suggested a smaller interindividual variability, so it was the best neuronal parameter for the detection of MS, followed by ganglion cell complex (GCC, combining RNFL + GCL + inner plexiform layer) from the macular area [**13**]. Furthermore, OCT scans have been linked to progressive retinal and brain atrophy [**14**,**15**]. According to a cohort research, monitoring peripapillary RNFL is crucial to predict the five-year risk of disability worsening in patients with MS [**14**,**16**]. Reduced retinal thickness has a correlation with clinical and paraclinical variables, such as visual function and MRI [**4**], and it is inversely correlated with the disease duration [**8**] and disability [**14**]. 

There are only a few OCT-A studies, but with heterogeneous results. In most studies, it was found that the vessel density (VD) in the superficial capillary plexus (SCP) in MS eyes is decreased compared to controls [**17**,**18**]. It can also be unaffected [**19**] or even increased, but only in MS patients without history of ON [**11**,**20**]. Regarding deep capillary plexus (DCP) in MS eyes, it can be decreased [**20**,**21**], unaffected [**22**], or increased [**11**] compared to controls. Regarding choroidal vascular network, it can be unaffected [**20**] or increased for MS patients without history of ON [**23**]. The causes of these OCT-A inconsistencies are unclear, however they might be linked to the selection of study population, such as including the different MS subtypes with or without history of ON, OCT-A data interpretation or maybe they are different stages in the pathophysiology of this disease. It is reported that the best OCT-A microvasculature parameter for the detection of MS is the SCP [**20**]. OCT-A can detect even the subclinical vascular changes [**17**], and the reduced retinal VD is correlated with Expanded Disability Status Scale (EDSS) [**10**], resulting that OCT-A parameters could be promising biomarkers for screening, diagnosis and monitoring of MS [**10**,**17**]. 

The detection of neurodegenerative changes using OCT and the detection of microvascular changes using OCT-A have allowed scientists to understand more about the pathophysiology of MS, but they have generally focused on using either structural thickness [**24**,**25**] or vascular characteristics [**17**,**19**] alone. Therefore, there is a lack of information on the combined impact of OCT and OCT-A parameters as potential biomarkers in MS.

## Conclusion

Using both structural and vascular characteristics could improve the diagnostic performance of MS and the risk prediction for disease progression in MS. However, validation studies are needed to establish that the OCT and OCT-A parameters could be used for the development of retinal biomarkers for screening, early diagnosis and monitoring the disease progression of MS, and for the development of potential future therapies that could slow or stop the course of this incurable disease. The aim of this literature review was to highlight the most current OCT and OCT-A data in MS and to serve as a resource for future studies.

**Conflict of interest statement**

The authors state no conflict of interest.

**Acknowledgements**

None.

**Sources of Funding**

None.

**Disclosures**

None.

## References

[1] McGinley MP, Goldschmidt CH, Rae-Grant AD (2021). Diagnosis and Treatment of Multiple Sclerosis: A Review. JAMA - Journal of the American Medical Association.

[2] Cennamo G, Carotenuto A, Montorio D, Petracca M, Moccia M, Melenzane A, Tranfa F, Lamberti A, Spiezia AL, Servillo G, De Angelis M, Petruzzo M, Criscuolo C, Lanzillo R, Brescia Morra V (2020). Peripapillary Vessel Density as Early Biomarker in Multiple Sclerosis. Front Neurol.

[3] Kashani AH, Asanad S, Chan JW, Singer MB, Zhang J, Sharifi M, Khansari MM, Abdolahi F, Shi Y, Biffi A, Chui H, Ringman JM (2021). Past, present and future role of retinal imaging in neurodegenerative disease. Progress in Retinal and Eye Research.

[4] Britze J, Frederiksen JL (2018). Optical coherence tomography in multiple sclerosis. Eye.

[5] Alonso R, Gonzalez-Moron D, Garcea O (2018). Optical coherence tomography as a biomarker of neurodegeneration in multiple sclerosis: A review. Multiple Sclerosis and Related Disorders.

[6] Greig EC, Duker JS, Waheed NK (2020). A practical guide to optical coherence tomography angiography interpretation. International Journal of Retina and Vitreous.

[7] Munk MR, Giannakaki-Zimmermann H, Berger L, Huf W, Ebneter A, Wolf S, Zinkernagel MS (2017). OCT-angiography: A qualitative and quantitative comparison. PLoS One.

[8] Chalkias IN, Bakirtzis C, Pirounides D, Boziki MK, Grigoriadis N (2022). Optical Coherence Tomography and Optical Coherence Tomography with Angiography in Multiple Sclerosis. Healthc.

[9] Thompson AJ, Banwell BL, Barkhof F, Carroll WM, Coetzee T, Comi G, Correale J, Fazekas F, Filippi M, Freedman K, Fujihara K, Galetta SL, Hartung HP, Kappos L, Lublin FD, Marrie RA, Miller AE, Miller DH, Montalban X, Mowry EM, Sorensen PS, Tintoré M, Traboulsee AL, Trojano M, Uitdehaag BMJ, Vukusic S, Waubant E, Weinshenker BG, Reingold SC, Cohen JA (2018). Diagnosis of multiple sclerosis: 2017 revisions of the McDonald criteria. The Lancet Neurology.

[10] Lanzillo R, Cennamo G, Criscuolo C, Carotenuto A, Velotti N, Sparnelli F, Cianflone A, Moccia M, Brescia Morra V (2018). Optical coherence tomography angiography retinal vascular network assessment in multiple sclerosis. Mult Scler J.

[11] Jiang H, Gameiro GR, Liu Y, Lin Y, Hernandez J, Deng Y, Gregori G, Delgado S, Wang J (2020). Visual Function and Disability Are Associated with Increased Retinal Volumetric Vessel Density in Patients with Multiple Sclerosis. Am J Ophthalmol.

[12] Petzold A, Balcer L, Calabresi PA, Costello F, Frohman T, Frohman E, Martinez-Lapiscina EH, Green A, Kardon R, Outteryck O, Paul F, Schippling S, Vermersch P, Villoslada P, Balk L, Aktas O, Albrecht P, Ashworth J, Asgari N, Black G, Boehringer D, Behbehani R, Benson L, Bermel R, Bernard J, Brandt A, Burton J, Calabresi P, Calkwood J, Cordano C, Courtney A, Cruz-Herranz A, Diem R, Daly A, Dollfus H, Fasser C, Finke C, Frederiksen J, Garcia-Martin E, Suárez IG, Pihl-Jensen G, Graves J, Havla J, Hemmer B, Huang SC, Imitola J, Jiang H, Keegan D, Kildebeck E, Klistorner A, Knier B, Kolbe S, Korn T, LeRoy B, Leocani L, Leroux D, Levin N, Liskova P, Lorenz B, Preiningerova JL, Martínez-Lapiscina EH, Mikolajczak J, Montalban X, Morrow M, Nolan R, Oberwahrenbrock T, Oertel FC, Oreja-Guevara C, Osborne B, Papadopoulou A, Ringelstein M, Saidha S, Sanchez-Dalmau B, Sastre-Garriga J, Shin R, Shuey N, Soelberg K, Toosy A, Torres R, Vidal-Jordana A, Waldman A, White O, Yeh A, Wong S, Zimmermann H (2017). Retinal layer segmentation in multiple sclerosis: a systematic review and meta-analysis. Lancet Neurol.

[13] Chua J, Bostan M, Li C, Sim YC, Bujor I, Wong D, Tan B, Yao X, Schwarzhans F, Garhöfer G, Fischer G, Vass C, Tiu C, Pirvulescu R, Popa-Cherecheanu A, Schmetterer L (2022). A multi-regression approach to improve optical coherence tomography diagnostic accuracy in multiple sclerosis patients without previous optic neuritis. NeuroImage Clin.

[14] Balıkçı A, Parmak Yener N, Seferoğlu M (2022). Optical Coherence Tomography and Optical Coherence Tomography Angiography Findings in Multiple Sclerosis Patients. Neuro-Ophthalmology.

[15] Abalo-Lojo JM, Limeres CC, Gómez MA, Baleato-González S, Cadarso-Suárez C, Capeáns-Tomé C, Gonzalez F (2014). Retinal nerve fiber layer thickness, brain atrophy, and disability in multiple sclerosis patients. J Neuro-Ophthalmology.

[16] Martinez-Lapiscina EH, Arnow S, Wilson JA, Saidha S, Preiningerova JL, Oberwahrenbrock T, Brandt AU, Pablo LE, Guerrieri S, Gonzalez I, Outteryck O, Mueller AK, Albrecht P, Chan W, Lukas S, Balk LJ, Fraser C, Frederiksen JL, Resto J, Frohman T, Cordano C, Zubizarreta I, Andorra M, Sanchez-Dalmau B, Saiz A, Bermel R, Klistorner A, Petzold A, Schippling S, Costello F, Aktas O, Vermersch P, Oreja-Guevara C, Comi G, Leocani L, Garcia-Martin E, Paul F, Havrdova E, Frohman E, Balcer LJ, Green AJ, Calabresi PA, Villoslada P (2016). Retinal thickness measured with optical coherence tomography and risk of disability worsening in multiple sclerosis: A cohort study. Lancet Neurol.

[17] Cordon B, Vilades E, Orduna E, Satue M, Perez-Velilla J, Sebastian B, Polo V, Larrosa JM, Pablo LE, Garcia-Martin E (2020). Angiography with optical coherence tomography as a biomarker in multiple sclerosis. PLoS One.

[18] Yilmaz H, Ersoy A, Icel E (2020). Assessments of vessel density and foveal avascular zone metrics in multiple sclerosis: an optical coherence tomography angiography study. Eye.

[19] Wang X, Jia Y, Spain R, Potsaid B, Liu JJ, Baumann B, Hornegger J, Fujimoto JG, Wu Q, Huang D (2014). Optical coherence tomography angiography of optic nerve head and parafovea in multiple sclerosis. Br J Ophthalmol.

[20] Bostan M, Chua J, Sim YC, Tan B, Bujor I, Wong D, Garhöfer G, Tiu C, Schmetterer L, Popa-Cherecheanu A (2022). Microvascular changes in the macular and parafoveal areas of multiple sclerosis patients without optic neuritis. Sci Rep.

[21] Khader S, Nawar A, Ghali A, Ghoneim A (2021). Evaluation of optical coherence tomography angiography findings in patients with multiple sclerosis. Indian J Ophthalmol.

[22] Ulusoy MO, Horasanlı B, Işık-Ulusoy S (2020). Optical coherence tomography angiography findings of multiple sclerosis with or without optic neuritis. Neurol Res.

[23] Farci R, Carta A, Cocco E, Frau J, Fossarello M, Diaz G (2020). Optical coherence tomography angiography in multiple sclerosis: A cross-sectional study. PLoS One.

[24] Cennamo G, Romano MR, Vecchio EC, Minervino C, Della Guardia C, Velotti N, Carotenuto A, Montella S, Orefice G, Cennamo G (2016). Anatomical and functional retinal changes in multiple sclerosis. Eye.

[25] Huang-Link YM, Fredrikson M, Link H (2015). Benign multiple sclerosis is associated with reduced tinning of the retinal nerve fiber and ganglion cell layers in non-optic-neuritis eyes. J Clin Neurol.

